# Molecular interactions at the surface of extracellular vesicles

**DOI:** 10.1007/s00281-018-0682-0

**Published:** 2018-04-16

**Authors:** Edit I. Buzás, Eszter Á. Tóth, Barbara W. Sódar, Katalin É. Szabó-Taylor

**Affiliations:** 1 Department of Genetics, Cell- and Immunobiology, Semmelweis University, Budapest, Hungary; 2 MTA-SE Immune-Proteogenomics Research Group, Budapest, Hungary

**Keywords:** Extracellular vesicle, Surface, Interactome, Extracellular matrix, Drug delivery

## Abstract

Extracellular vesicles such as exosomes, microvesicles, apoptotic bodies, and large oncosomes have been shown to participate in a wide variety of biological processes and are currently under intense investigation in many different fields of biomedicine. One of the key features of extracellular vesicles is that they have relatively large surface compared to their volume. Some extracellular vesicle surface molecules are shared with those of the plasma membrane of the releasing cell, while other molecules are characteristic for extracellular vesicular surfaces. Besides proteins, lipids, glycans, and nucleic acids are also players of extracellular vesicle surface interactions. Being secreted and present in high number in biological samples, collectively extracellular vesicles represent a uniquely large interactive surface area which can establish contacts both with cells and with molecules in the extracellular microenvironment. Here, we provide a brief overview of known components of the extracellular vesicle surface interactome and highlight some already established roles of the extracellular vesicle surface interactions in different biological processes in health and disease.

## Introduction

Extracellular vesicles (EVs) are membrane-enclosed heterogeneous structures that are secreted by all cells [1] and have many different physiological and pathophysiological roles [2]. They include small EVs of endosomal origin (exosomes) as well as plasma membrane-derived intermediate-sized (100–1000 nm) microvesicles, and large sized (> 1 μm) apoptotic bodies and large oncosomes [3, 4]. In the past few years, EVs attracted rapidly growing scientific interest from various fields of biomedicine.

Surface molecules of EVs are of critical functional significance as they (i) establish connections with the surrounding micro milieu and with cells, (ii) determine EV mobility, (iii) mediate cellular uptake, (iv) affect immune recognition of EVs (also via posttranslational modifications) by the innate and adaptive immune systems, and (v) may represent effector molecules (such as FasL). On the other hand, from a researcher’s perspective, they enable identification, affinity isolation, and molecular classification of EVs and EV subtypes, and enable the use of EVs as biomarkers.

Here, we overview EV surface interactions with the surrounding microenvironment (extracellular matrix (ECM) molecules or components of the blood plasma) and with cells and provide examples for the functional relevance of the surface interactions of EVs.

## Evidences for exofacial localization of EV proteins as partners in EV surface interactions

When considering EV surface interactions, it is of crucial importance to define EV molecules with exofacial topology that can serve as interaction partners. EV surface molecules are identified by immunolabeling (immunogold electron microscopy, flow cytometry or immunochemistry using confocal or super resolution microscopy). These widely used approaches enabled identification of “canonical” EV surface proteins including tetraspanins (*CD9*, *CD63*, and *CD81*), integrins (*ITG*), cell adhesion molecules (*CAM*), and growth factor receptors [5]. The presence of these molecules has been confirmed by many different laboratories.

Mass spectrometry (MS)-based proteomic characterization has proven to be a very efficient and widely used tool to characterize EVs. This approach was first used by Thery et al. [6] for the characterization of exosomes followed by many other studies over the years. These proteomic data are also publicly available from databases (Exocarta, EVpedia, and Vesiclepedia) (http://student4.postech.ac.kr/evpedia2_xe/xe/, http://www.exocarta.org/, http://www.microvesicles.org/). However, MS does not enable identification of the precise topology of EV proteins. Possible membrane defects due to centrifuge-based EV isolation procedures or the occurrence of inverted vesicles may enable labeled antibodies to recognize internal cargo molecules of EVs making the distinction between EV surfaces and internal cargo proteins challenging. This possibility cannot be completely excluded even when using, e.g., antibody-coated EV arrays [7].

Recently, a combination of proteinase treatment and subsequent biotinylation, a strategy known from studying cellular membrane proteins, has been suggested for the study of luminal and surface-accessible EV cargo [8]. Even with this approach, it cannot be determined whether the surface-accessible EV proteins were present already at the time of EV production or they were subsequently acquired from conditioned media or biological fluids.

Strong evidence for EV surface localization of certain molecules comes from the ability to target the putative protein (or other molecule) for affinity isolation of EVs. Anti-EpCAM and anti-A33 antibodies were used for immunocapture of colon cancer-derived exosomes [9]. Similarly, anti-tetraspanin (antiCD63, CD9 and CD81) antibodies can be used for immunoisolation of EVs [3]. Immune electron microscopy revealed that hsp70 is localized on the surface of exosomes [10], and a synthetic peptide (Vn96) with high affinity for heat shock proteins has proven useful for affinity enrichment of cancer EVs [11–13]. Furthermore, EVs can be isolated by heparin affinity purification. Suggested heparin-binding proteins on EVs include histones, heat shock proteins, and annexin; however, definite interacting ligand(s) have not been determined yet [14]. Of note, not only proteins but also other surface molecules are targeted for EV affinity capture. As an example, the recently identified phosphatidyl serine (PS) receptor TIM4 [15] was found efficient in capturing PS-exposing EVs [16].

For immunodetection of EV surface molecules, dot scan (antibody microarray) has been used recently. It showed moderate/high levels of CD19, CD5, CD31, CD44, CD55, CD62Lm, CD82, HLA-A, B, and C and low levels of CD21, CD49c, and CD63 on EVs. The authors proposed these EV surface molecules as a diagnostic signature for chronic lymphocytic leukemia [17]. Furthermore, surface plasmon resonance (SPR) has been used recently for the simultaneous detection of both EV and cancer markers on exosomes from breast cancer cells [18]. Moreover, exosome “surfaceome” profiling was carried out by an initial MS testing EVs secreted by 13 pancreatic ductal adenocarcinoma cell lines and 2 non-neoplastic cell lines. MS was followed by identification of candidate biomarkers and validation by an immunocapture pulldown assay. In this assay, a multiplexed panel of antibodies was used that included anti-CLDN4, EPCAM, CD151, LGALS3BP, HIST2H2BE, and HIST2H2BF antibodies for the enrichment of tumor-specific exosomes for subsequent studies [19].

Numerous pieces of evidence suggest that surface molecules on EVs determine the uptake and biological functions of EVs. As one example, blockade of exosome surface SIRPα (CD47) was shown to be effective in increasing cancer cell phagocytosis [20].

## Interaction of EVs with the plasma membrane of cells

Surface interactions of EVs with the plasma membrane are of outstanding importance since such interactions mediate binding of EVs to cells resulting in signal transduction or uptake of EVs by cells. It is now established that EV-target cell interactions involve tetraspanins, integrins, ECM proteins, immunoglobulin superfamily members, proteoglycans, and lectins [21, 22]. Details of EV docking and entry to cells are not in the focus of this review, as these interactions have recently been reviewed elsewhere [21, 22]. To illustrate the outstanding functional significance of the interaction of EV surface molecules with those of the plasma membrane, here, we only refer to the plethora of EV-immune cell interactions including cell-free antigen presentation by EVs [23], Fas ligand or TRAIL-mediated cell death induction by EVs [24–26], or the transfer of immune checkpoint molecules (PD1, PDL-1) by EVs [27].

**Fig. 1:**
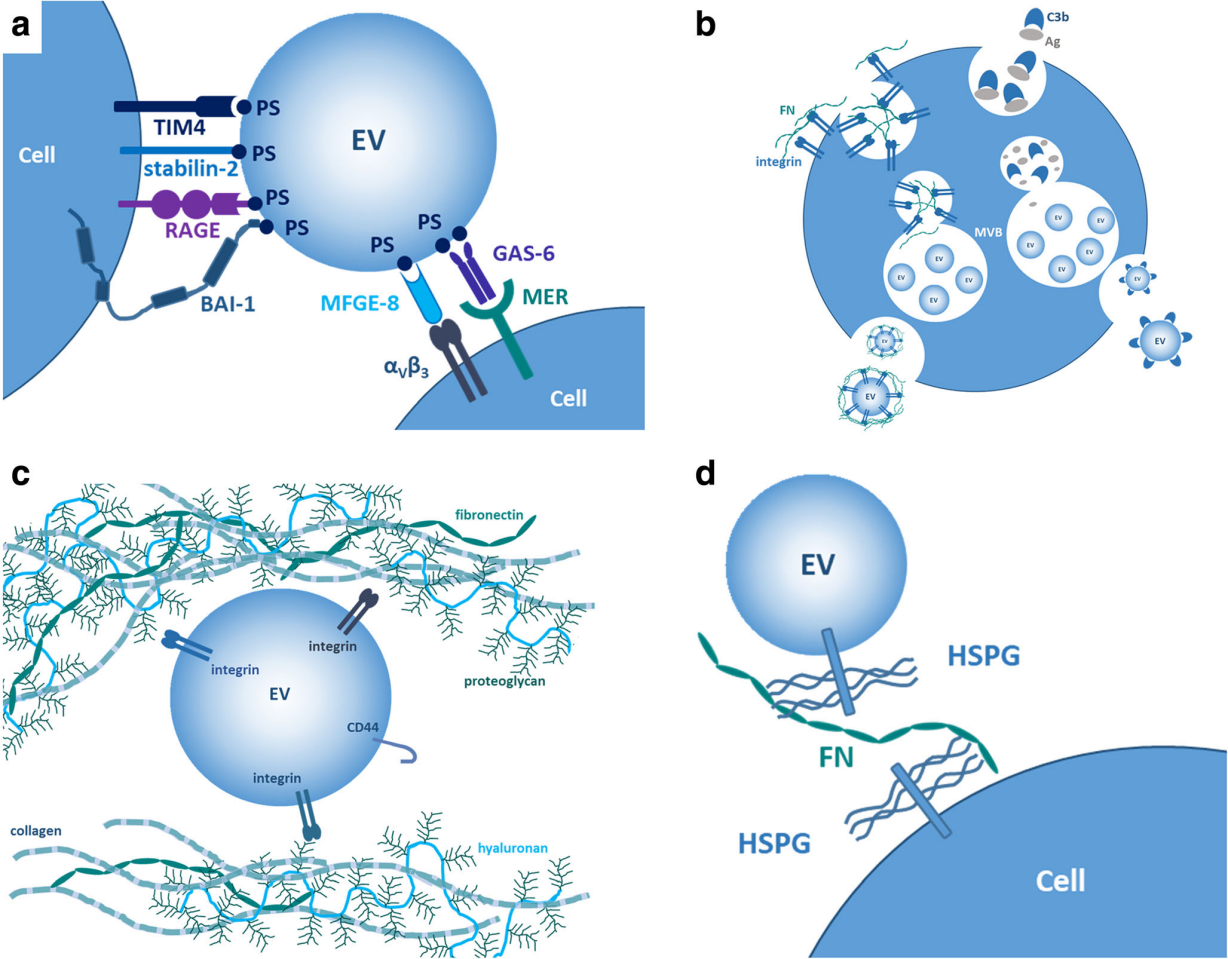
Examples for EV surface interactions with the plasma membrane and components of the extracellular matrix. **a** One of the best characterized interactions between the plasma membrane and the surface of EVs is mediated by proteins that recognize externalized phosphatidyl serine (PS) on EVs. Direct interactions with PS include those with TIM4, stabilin-2, RAGE, or BAI-1. Indirect interactions include those between the PS-binding MFGE-8 and α_v_β_3_ integrin as well as the PS-binder GAS-6 and the MER tyrosine kinase on the cell. **b** Endocytosis of fibronectin (FN) or C3b complement protein is followed by an association of these molecules with intraluminal vesicles within MVBs followed by secretion of exosomes with surface-associated FN or C3b. **c** Interaction of EVs with ECM is mediated by integrins or CD44. **d** FN forms a bridge between HSPGs present on both EV surface and plasma membrane, and mediates EV uptake by cells

Here, we also point out the significance of externalization (translocation to the outer leaflet of a phospholipid bilayer) of phosphatidyl serine (PS), a characteristic feature of many EVs. The negatively charged, surface-exposed phospholipid PS is recognized by numerous plasma membrane receptors either directly or indirectly, via bridging proteins. Direct PS sensing receptors include the previously mentioned TIM4 [15], the receptor for advanced glycation end products, RAGE [28], brain-specific angiogenesis inhibitor 1 Bai-1 [29], and stabilin-2 [30]. Indirect PS recognition and subsequent uptake is mediated by milk fat globule-EGF factor 8, MFGE8 [31] which forms a molecular bridge between PS and plasma membrane integrins (such as α_v_β_3_) [31] (Fig. 1a).Of note, MFGE8 is not only detectable on the surface of exosomes [32], but it is also secreted by cells as an EV-MFGE8 complex [33]. This EV-protein complex secretion is similar to secretion EV-integrin-FN and EV-C3b complexes (Fig. 1b).

Of note, similar indirect recognition of PS is also described in the case of PS recognized by the growth arrest-specific protein 6, Gas6. The PS-Gas6 complex was shown to activate TAM family member MER tyrosine kinases on the surface of macrophages triggering uptake and inducing an anti-inflammatory phenotype [34].

Until now, most studies investigated PS–plasma membrane receptor interactions focusing on the uptake of apoptotic cell-derived vesicles. This is explained by the fact that apoptosis has been long known to be accompanied by PS externalization. However, given that externalized PS is characteristic for many EV surfaces, and annexin V is used broadly to detect EVs, it seems plausible that PS-mediated interactions with the plasma membrane govern the binding and uptake of non-apoptotic EVs as well. Indeed, there are accumulating pieces of evidence that show PS-mediated EV uptake or signaling in the case of non-apoptotic vesicles also [35, 36].

## Interaction of EV surfaces with the extracellular matrix: extracellular binding or re-cycling?

It is an important question whether EVs secreted by cells of tissues rich in extracellular matrix (ECM) such as connective tissue, interact with matrix molecules. Accumulating pieces of evidence suggest that indeed such interactions exist and their significance is increasingly recognized. It may seem intuitive that EV surfaces interact with the ECM components upon secretion, once being surrounded by the macromolecular ECM milieu. This may predict that EV membrane deposition of matrix molecules results from binding of these molecules onto EV surfaces extracellularly. Although newly secreted EVs evidently establish interactions with ECM molecules in tissues and body fluids (Fig. 1c), there seems to be another mechanism, which may explain the presence of certain ECM molecules on the surface of EVs. It has been proposed recently that cells endocytose ECM molecules and re-secrete them on the exofacial surface of EVs (exosomes) [37] (Fig. 1b). This continuous endocytosis and re-secretion of ECM components guarantees an abundant source of ECM-carrying EVs, which may play an important role in cell migration. Such endocytosis and EV-associated re-secretions has been recently demonstrated in the case of fibronectin (FN)–integrin complexes. FN is endocytosed in association with integrins, it is then targeted to MVB, where it binds to the surface of intraluminal vesicles in correct topology to interact with both the cell surface and other ECM molecules (e.g. collagen fibers) [37].

Kowal et al. used immuno-isolated EVs by CD9, CD63, and CD81-specific antibodies. The authors have demonstrated the existence of a subtype of small EVs (sEVs) that the authors referred to as “dense sEVs” which carried FN, complement, prothrombin, and serum albumin, while another subpopulation of sEVs (“light sEVs”) did not carry any of these molecules on its surface [3]. Whether dense sEVs acquired their ECM coat from the conditioned medium of the cells upon secretion or were secreted with surface-bound ECM molecules, was not investigated in this study.

## Fibronectin

One of the most extensively studied ECM molecules with respect to surface interaction with EVs is FN. FN binds multiple integrins. It has been shown that reticulocyte maturation is accompanied by release of EVs carrying α4β1 integrin (Very Late Antigen-4, VLA4) by which EVs were shown to bind to FN [38]. Myeloma-derived EVs (exosomes) were found to carry FN on their surface [39]. This exofacially bound FN could interact with cell surface heparan sulfate (through its Hep-II domain). The authors showed that FN could simultaneously bind to heparan sulfate proteoglycans both on the exosomal and the plasma membrane surfaces thereby facilitating cellular uptake of EVs [39] (Fig. 1d).

There are multiple evidences that beyond facilitating cell binding and cellular uptake, there are other functional consequences of EV-association of FN. A striking function of FN on EVs is related to cellular motility. As described above, FN bound to integrins on exosomes was shown to promote directional cancer cell movement by reinforcing transient polarization states and adhesion assembly [37]. Furthermore, exosomal FN was shown to induce IL-1β expression by macrophages [40]. We have shown recently that DNA present on the surface of small EVs secreted by stressed cells facilitated interaction of EVs with FN [41]. Finally, FN on circulating EVs in liquid biopsy samples of breast cancer patients samples was suggested to be a promising cancer biomarker [42].

## Glycosaminoglycans (GAGs) and proteoglycans

Heparan sulfate proteoglycans (HSPGs) are abundant glycoproteins having a core protein to which one or more heparan sulfate (HS) glycosaminoglycan (GAG) chains are attached covalently. Membrane-bound HSPGs include syndecans and glypicans. Interestingly, syndecans and glypicans are present both on the plasma membrane of the EV-releasing cells and the membrane of EVs. Cancer cell-surface HSPGs of the syndecan and glypican types were shown to mediate internalization of EVs [43]. This process was readily inhibited by free heparan sulfate. Importantly, the same study demonstrated sorting of HSPGs to EVs (exosomes) [43]. As we mentioned earlier, by forming a bridge between EV and plasma membrane HSPGs, FN was shown to mediate EV uptake [39].

Recently, the presence of glypican 1 associated with exosomes was demonstrated by different groups [44–46]. Its proposed exploitation as a biomarker of pancreatic cancer is currently under investigation.

Among other ECM molecules, hyaluronan (HA) synthesis was shown to be associated with the shedding of HA-coated EVs by human mesenchymal stem cells (Fig. 1d). HA coating on EVs was proposed to (i) contribute to HA-mediated tissue regeneration, (ii) regulate interactions of EVs with target cells, and (iii) play a role in ECM remodeling [47]. Not only HA but also the HA receptor CD44 is associated with EV surfaces. Ovarian cancer cell invasion was shown to be supported by exosomal transfer of CD44 to peritoneal mesothelial cells [48]. CD44 was also identified as a component of the cancer cell-derived circulating EV-specific diagnostic signature [17] and was recently shown to serve as one of the diagnostic and prognostic exosomal biomarkers of breast cancer [49]. Interestingly, transcripts of CD44 are also carried horizontally as internal cargo in human mesenchymal stem cell-derived HA-coated EVs [47].

## The role of integrins in EV-ECM interactions

Integrins represent a group of transmembrane receptors that play a role in cell-ECM adhesion. Known integrin ligands in the ECM include molecules such as fibronectin, collagen, vitronectin, and laminin. Numerous pieces of evidence support that among EV surface adhesion molecules, integrins play a distinguished role. Tumor EVs (exosomes) can promote cancer progression by transferring integrin transcripts horizontally and by selecting metastatic sites as reviewed recently [50]. Tumor-derived EV (exosome) integrins α_6_β_4_ and α_6_β_1_ correlated with the development of lung metastasis, while exosomal integrin α_v_β_5_ was associated with liver metastasis [51]. This important observation suggests that there is a potential of EVs to predict metastatic sites of tumors based on their surface integrins.

## EVs and the blood plasma

### Immunoglobulins

**Fig. 2:**
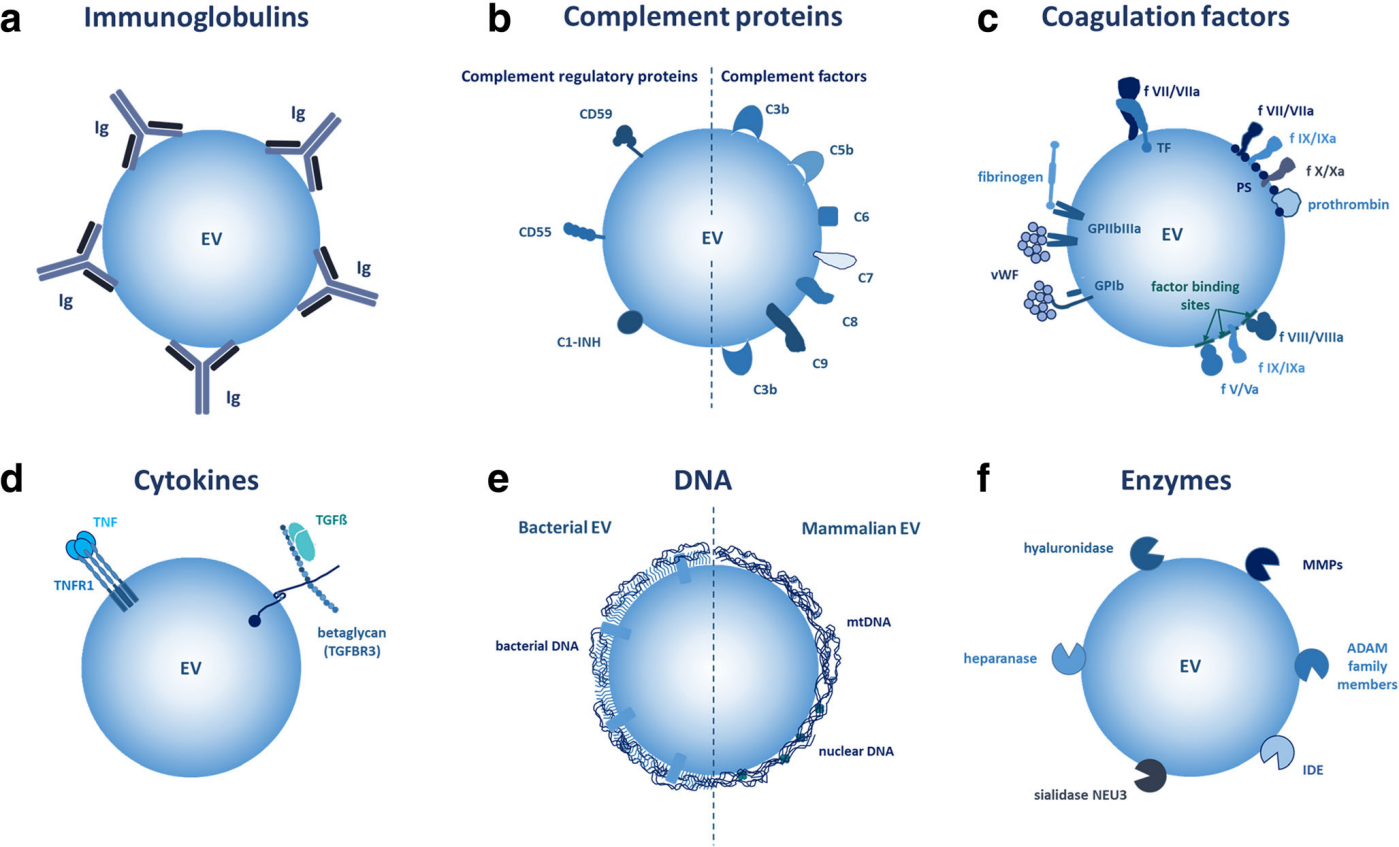
Examples for EV surface-associated molecules. **a** Antibody binding to EVs has been demonstrated, e.g., in numerous autoimmune diseases. **b** Both complement factors and complement regulatory proteins have been shown to associate with EV surfaces. **c** On EVs from blood plasma, different coagulation factors are also identified. **d** EV-associated cytokines include TNF bound to TNF receptor as well as TGFβ bound to TGFβR3 (betaglycan) on EV surfaces. **e** Both bacterial and mammalian EVs have been demonstrated to carry surface-associated DNA and DNA-binding proteins. In the case of mammalian EVs, both mitochondrial and nuclear DNA were found on EV surfaces. **f** A surprisingly large variety of EV surface enzymes were identified that can bind and cleave protein or glycan substrates of the EV microenvironment

The association of EVs with plasma factors, notably immunoglobulins and complement factors (Fig. 2a, b), is best described concerning the spectrum of autoimmune rheumatological diseases. Systemic lupus erythematosus (SLE) and rheumatoid arthritis (RA) are autoimmune diseases with a significant type III hypersensitivity component meaning that immune complexes and complement activation contribute to the disease pathology.

EVs have been known to associate with autoantibodies in several autoimmune diseases, forming pro-inflammatory immune complexes contributing to disease pathology as we reviewed recently [52]. In RA synovial fluid, platelet EVs display autoantigens and form immune complexes, which potently activate neutrophils thereby perpetuating inflammation [53]. SLE is an immune complex disease where disease symptoms arise due to the reduced clearance of immune complexes, which leads to complement-mediated inflammation. EVs also associate with immunoglobulins and enhance the formation of such pathological immune complexes in SLE [54]. A recent study showed that distinct subpopulations of EVs harboring immunoglobulins were associated with distinct clinical characteristics of SLE and may therefore serve as biomarkers in future [55].

Autoimmune phenomena can also arise due to autoantibodies produced against nucleic acids. EV-associated chromatin is normally digested off by DNAse1L3. The loss of this mechanism can lead to the formation of autoantibodies which in turn can cause autoimmunity [56].

### Complement

It was demonstrated that complement activation occurs on platelet-derived microvesicles (also referred to as microparticles). Complement proteins (C3b and C5b-9) were shown to deposit on the surface of platelet-derived EVs exposed to blood plasma. Of note, not only complement proteins but also complement regulatory proteins (C1-INH, CD55, and CD59) were present on platelet EVs. The authors proposed that these EVs may present concentrated activated complement components to targets in the blood vessels [57].

Complement components have a major role in the clearance of apoptotic cells. In SLE, the mechanism of apoptotic cell clearance is damaged which leads to the disease symptoms of widespread inflammation due to chronic complement activation. Complement components associated with EVs and an altered binding of C3 components to EVs were observed in SLE even though there was no difference in the concentration of EVs between SLE patients and healthy subjects. SLE patients had higher levels of C3d-positive EVs and lower levels of C3b and C3ib-positive EVs. Since the latter components opsonize cells and EVs for phagocytosis, this difference could also contribute to chronic inflammation [58]. Association of complement factors with EVs in different types of renal disease has been extensively reviewed in [59].

It appears that in autoimmune and renal diseases, binding of different complement factors to EVs is preferential. Similarly, the attachment of complement factors, immunoglobulins, and other serum components to artificial particles depends on the particles’ surface chemistry. Differential binding of such plasma components has an influence on the adjuvant properties of the particles and thus has an influence on the use of these particles in vaccine delivery. Importantly, complement factors were necessary for the uptake of the artificial particles by antigen presenting cells via complement receptor 3 in mice [60].

EV-associated complement proteins may not only directly attach onto EVs upon exposure to blood plasma. C3 fragments were detected by immune electron microscopy in MVBs on the surface of intraluminal vesicles [60]. This may represent another example for endocytic uptake and exosomal re-secretion of an extracellular protein. These C3b-coated EVs were suggested to have an immunomodulatory role by enhancing the antigen presentation [60] (Fig. 2b).

### Association of coagulation factors with EVs

Early evidence for procoagulant surfaces in platelet-free blood plasma was published by Wolf and collegues and was described as “platelet dust” back in 1967 [61]. Since then, a high number of studies confirmed that platelet-derived EVs, highly abundant in blood plasma, indeed have procoagulant properties. Furthermore, non-platelet EVs such as tumor derived vesicles [62] proved to affect hemostasis partially by assembling factors of coagulation on their surface in the blood plasma. The most extensively studied two components of EVs in coagulation are phosphatidylserine (PS) and tissue factor (TF) (Fig. 2c).

As a high percentage of platelet-derived MVs bear the anionic phospholipid PS on their outer membrane, they facilitate the assembly of several proteins of the coagulation cascade. These proteins contain positively charged γ-carboxyglutamic acid domains to which PS can bind with electrostatic interaction. These factors include VII, IX, X, and prothrombin [63]. Underlining the importance of PS-positive EV formation in hemostasis, patients suffering from a rare bleeding disorder (Scott syndrome) were found to have reduced floppase activity resulting in faulty PS externalization and reduced microparticle shedding [64].

Also, TF, a transmembrane receptor of factor VII/VIIa, can be present on MVs (vesicles often referred to as microparticles, MPs in coagulation studies). The elevated activity levels of this protein have been detected in various diseases (such as in acute liver injury, cirrhosis, urinary tract infection, endotoxemia, influenza, cancer, and related thromboembolism [65]). The platelet origin of TF on platelet MVs and the overall relevance of TF-bearing platelet MVs have been questioned by several authors [66–68]. However, the role of TF positive EVs irrespective whether they originate form platelets, tumor cells, endothelial cells, or leukocytes is clear in various hemostatic diseases and diseases tipically presenting with thrombembolic complications (as detailed in the comprehensive review by Owens and Mackman [63]). The presence of TF on EVs makes the presence of its specific inhibitor molecule, tissue factor pathway inhibitor (TFPI), also probable [63].

In addition to assembling factors that initiate the coagulation cascade, platelets and their MPs also can present specific binding sites for factors V, IX, and VIII [69–71]. Indeed, these binding sites can be found concentrated on MPs relative to platelets. In the case of factor Va and VIIIa, a 10-fold, while in the case of factor IXa, a 2-fold concentration of factor binding sites was observed in the above mentioned studies [69–71]. Another factor, von Willebrand Factor (vWF), an interaction partner of both glycoproteins GPIb and GPIIbIIIa, was found to be attached to platelet- and also to endothelial cell-derived EVs [72, 73]. Together, the accumulation of PS and of other coagulation factor binding sites enables the surface of platelet MPs to enhance coagulation approximately 50–100-fold as compared to platelets [74].

Interestingly, depending on what stimuli the parent cell recieved, MPs may bear different surface molecules resulting in different binding features. For instance, platelets activated with thrombin or collagen were found to shed MPs exposing GPIIbIIIa complexes binding fibrinogen, while those activated with C5b-9 shed non-GPIIbIIIa-exposing MPs [70].

Although the effect of platelet-derived microvesicles has been studied most widely, it is important to note that activated platelets also secrete exosomes [75]. However, their association with coagulation factors in plasma is questionable, as they, if at all, bear very low levels of PS [76]. Also, it is controversial whether plasma exosomes of different cellular origin bear TF [77].

### Association of EVs with lipoproteins

Isolation of EVs from human blood plasma or serum is often confounded by the co-isolated lipoproteins [78–81]. Moreover, antibody-mediated depletion of lipoproteins [82] and lipoprotein apheresis [83] both resulted in loss of EV content as well. On the other hand, MS analysis of VLDL and LDL particles purified from human blood plasma revealed the presence of EV proteins (CD14, LDL-receptor, HLA class I molecules, and protein S100-A8) in these isolates [84]. Taken together, these data suggest that beyond the shared physiological parameters, there might be an association between lipoproteins and EVs as well. In vitro association has already been demonstrated by transmission electron microscopy [80]. However, experimental data are not available yet in support of an in vivo association of EVs and lipoproteins. Exchange of the protein and lipid content between lipoproteins is an established phenomenon [85–88]. Exchange of ApoE between lipoproteins and hepatitis C virus lipoviral particles has been also described [89]. Moreover, in vitro SR-B1-dependent transfer of a fluorescent phospholipid from engineered HDL nanoparticles to exosomes was also reported [90]. Finally, ApoE has been implicated in amyloid formation of pigment cells [91], and it has been shown that in these cells, ApoE associates with intraluminal vesicles and is secreted on the surface of exosomes [92].

### Further blood plasma proteins associated with the surface of circulating EVs

Beside the known role of EVs as carriers of luminal cargo, EVs may also carry a significant surface cargo. Technically challenging to investigate, so far, very little is known about the externally adsorbed proteins. It is likely that the external cargo of EVs is at least partly acquired in body liquids after the EVs have been shed. As an example, blood plasma-derived EVs commonly carry substantial amounts of albumin [93]. In line with this, proteomics data in EV databases (http://student4.postech.ac.kr/evpedia2_xe/xe/, http://www.exocarta.org/, http://www.microvesicles.org/), [94–96] show that blood plasma-derived EVs co-isolate with numerous blood plasma proteins. Given the known presence of integrins and HSPGs on EVs, integrin ligands and heparin binding proteins are evident potential partners to establish interactions on the surface of circulating EVs. Furthermore, phosphatidyl serine binding proteins (such as MFGE8) and glycan binding galectins are obvious interaction partners of EVs in the circulation. Systemic analysis of EVs surface interactions with blood plasma proteins is still lacking.

### Association of EVs with cytokines/chemokines

An increasing number of data support that EVs are capable of carrying various cytokines [2]. In most instances, these cytokines are carried in EVs as part of the internal cargo. However, it was shown that EVs carry a full-length 55-kDa tumor necrosis factor receptor 1 (TNFR1). Importantly, it was demonstrated by the authors that HUVEC-derived exosomes carried bound TNF [97] (Fig. 2d).

In addition, TGF beta was shown to be associated with the cell-surface chondroitin sulfate/heparan sulfate proteoglycan betaglycan (also referred to as transforming growth factor beta receptor III, TGFBR3) on the surface of cancer cell-derived exosomes. Although the authors found that the kinetics and magnitude of biological response were similar irrespective if they used soluble or EV-associated TGF beta, there were some qualitative differences in the elicited cellular responses [98] (Fig. 2d). Although it is tempting to hypothesize that additional cytokines (including chemokines) may be carried on the surface of EVs in association with EV surface proteoglycans, a systemic analysis of this question has not been performed yet.

### DNA associated with the surfaces of EVs

In bacteria, outer membrane vesicle (OMV)-associated DNA has been shown to mediate inter- and intra-species horizontal gene transfer by carrying antibiotic resistance genes and virulence factor [99–101], and participating in the establishment of bacterial biofilms [102, 103]. Recently, it was also reported that OMV-associated DNA was found predominantly on the outer surface of OMVs [104].

In mammalian systems, most studies so far focused on DNA encapsulated in EVs as an internal cargo, and only very few reports investigated the EV surface-associated DNase-sensitive DNA. Of note, these studies drew attention towards the potential of EV surface-associated DNA in horizontal gene transfer [105], induction of autoimmunity [56], and cellular uptake [106]. Recently, we have shown that antibiotic-exposed cells undergoing genotoxic shock secreted small EVs (exosomes) with surface-associated DNA which was predominantly mitochondrial DNA [41]. The amount of this DNA was not enhanced by induction of apoptosis of the EV-releasing cells. As mentioned earlier, exosome surface-associated DNA was capable of mediating EV binding to FN [41] (Fig. 2e).

### Enzymes associated with EV surfaces

Several pieces of evidence support the presence and activity of EV-associated enzymes as reviewed recently [107]. Thus, enzymes do not only represent components of the EV internal cargo but are also characterized by active exofacial enzymes. These include both proteases MT1-MMP (MMP-14) [108], ADAM17 [109], insulin-degrading enzyme (IDE) (insulin-like), and EV surface-associated glycosidases such as sialidase NEU3 [110, 111] and heparanase [112]. Importantly, EV surface-associated proteases and glycosidases may exert their function in concert with one another in matrix degradation. In addition, flow cytometry of isolated EVs bound to latex beads demonstrated the presence of multiple other enzymes (MMPs-2, -3, -9, -13, -14, ADAM-10, ADAM-17, ADAMTS-5, ADAMTS-8, uPAR, and hyaluronidase) [113]. EV-associated enzymes may (i) facilitate cell and EV mobility by degrading ECM macromolecules as substrates, (ii) release bound growth factors or chemokines, and (iii) destruct amyloid β plaques [114] (Fig. 2f).

### EV surface-associated thiols

Thiol interactions are relevant both in the release and uptake of EVs, and it is highly likely that the content and composition of exofacial thiols has a vast influence on the interactions of EVs with their environment including macromolecules. The total surface thiol content of EVs can also be utilized for labeling purposes [115]. Plasma-derived and tissue culture-derived EVs can equally be labeled by thiol-reactive fluorescent reagents. However, it is important to be aware that contaminating plasma proteins interfere with such labeling and therefore, a dual labeling protocol of EV thiols is preferable from plasma samples [115]. Certain plasma proteins, like albumin, have a reactive thiol moiety [116], and interactions with EVs may take place via thiol interactions. In particular, in the case of albumin, it seems feasible that EVs form part of the “albuminome” potentially extending their half-life in the circulation via interacting with albumin. Albumin certainly appears among the molecules associated with EVs as discussed above. Redox regulation of cellular surface molecules is an emerging factor affecting cellular functions. Importantly, adhesion molecules such as integrins underlie redox regulation. Reducing the α4-integrin by *N*-acetyl-cysteine leads to increased FN adhesion and cellular aggregation of Jurkat cells [117]. Similar redox regulation may be relevant in EV biology. EV-associated integrin regulation is of particular interest, since distinct expression pattern of integrins on EVs is responsible for organotropism in cancer metastasis [51]. Therefore, redox regulation of exofacial molecules on EVs is likely to affect their functions.

Several thiol-reactive antioxidants are present in the plasma, notably certain members of the thioredoxin family such as peroxiredoxins 1, 2, and 4 and different forms of thioredoxin [118]. These thiol-reactive antioxidants are also known to appear on the surface of cells [118, 119]. Therefore, it is to be expected that these molecules also appear on the surface of EVs either as membrane proteins or plasma proteins associating with the EVs. Indeed, peroxiredoxin 2 was present on the surface of EVs [120, 121], and peroxiredoxin1 (Prdx1)-positive EVs were elevated in rheumatoid arthritis (RA) patient plasma compared with healthy controls which may be a marker of inflammation [115]. Since Prdx 1 is also present as a free protein in plasma, it may be released together with EVs or it associates with EVs after release, in the plasma. Protein disulfide isomerase is thought to be responsible for regulating cell surface thiols [122] and associated with EV surface, and it also seems to activate platelets [123].

## Conclusions

The relatively large surface to volume ratio of EVs enables highly efficient surface interactions of these structures with cells and extracellular molecules. Such surface interactions have outstanding importance since they determine the fate of EVs by targeting them to the plasma membrane of cells or to certain tissues. One of the most exciting aspects of EV surface interactions is that they can be tailored by engineering the EV-releasing cells [124, 125]. This way, by having the designed EV-producing cells, one may achieve to produce EVs with specific targeting molecules on their surface and thus may be able to alter the biodistribution of EVs used as drug delivery systems. Feasibility of this approach was first demonstrated when siRNA was successfully delivered to murine brain upon systemic administration of exosomes carrying a brain-targeting peptide [126]. This initial proof-of-concept study has been followed by several subsequent works in which designer EVs were specifically targeted to tissues or cells [125]. The potential of designing targeted EVs for drug delivery, and the known ability of exosomes to cross blood tissue barriers such as blood brain barrier, underlies the significance in emerging EV-based therapies including gene therapy.

Another aspect of EV surface interactions is that due to technical limitations, it is not feasible to perform comprehensive analysis of EVs in situ in living tissues. Considering the interactions of EVs both with cells and with molecules of the microenvironment, there is an urgent need for much more complex systems to model EV surface interactions.

Recognizing the complexity of EV surface interactions, we should change our way of thinking about EVs as “pure” membrane vesicles. We should rather consider EV surface interactome in our experimental design since EV surface-associated molecules can hinder those already present on the EV surface, resulting in unexpected outcomes of both analysis and isolation of EVs.

### Grant support

This work was supported by the National Research, Development and Innovation Office NKFIH, Hungary, OTKA11958, OTKA120237, NVKP_16-1-2016-0017, Ministry for National Economy of Hungary VEKOP-2.3.2-16-2016-00002, and VEKOP-2.3.315201600016.
